# Heterogeneous Distribution of Phospholipid Molecular Species in the Surface Culture of *Flammulina velutipes*: New Facts about Lipids Containing α-Linolenic Fatty Acid

**DOI:** 10.3390/jof9010102

**Published:** 2023-01-12

**Authors:** Svetlana V. Senik, Bairta S. Manzhieva, Liliya G. Maloshenok, Evgeny B. Serebryakov, Sergey A. Bruskin, Ekaterina R. Kotlova

**Affiliations:** 1 Komarov Botanical Institute of the Russian Academy of Sciences, 197376 St. Petersburg, Russia; 2 Vavilov Institute of General Genetics of the Russian Academy of Sciences, 117971 Moscow, Russia; 3 Chemical Analysis and Materials Research Center, St. Petersburg State University, 198504 St. Petersburg, Russia

**Keywords:** phospholipids, molecular species of phosphatidylcholine, α-linolenic fatty acid, basidiomycete, fungi

## Abstract

Mycelial fungi grow as colonies consisting of polar growing hyphae, developing radially from spore or inoculum. Over time, the colony develops, hyphae are subject to various exogenous or endogenous stimuli, and mycelium becomes heterogeneous in growth, gene expression, biosynthesis, and secretion of proteins and metabolites. Although the biochemical and molecular mechanisms of mycelium heterogeneity have been the subject of many studies, the role of lipids in colony development and zonality is still not understood. This work was undertaken to extend our knowledge of mycelium heterogeneity and to answer the question of how different lipid molecular species are distributed in the surface colony of the basidial fungus *Flammulina velutipes* and how this distribution correlates with its morphology. The heterogeneity in the lipid metabolism and lipid composition of the fungal mycelium was demonstrated. According to the real-time PCR and LC-MS/MS results, the expression of genes of PC metabolism, accumulation of phospholipid classes, and degree of unsaturation of PC and PE increased in the direction from the center to the periphery of the colony. The peripheral zone of the colony was characterized by a higher value of the PC/PE ratio and a higher level of phospholipids esterified by linolenic acid. Considering that the synthesis of phospholipids in fungi occurs in different ways, we also conducted experiments with deuterium-labeled phospholipid precursors and found out that the Kennedy pathway is the predominant route for PC biosynthesis in *F. velutipes*. The zonal differences in gene expression and lipid composition can be explained by the participation of membrane lipids in polar growth maintenance and regulation.

## 1. Introduction

The colony of mycelial fungus is a network of polar growing hyphae, developing radially from spore or inoculum. In the course of development, the growth of vegetative hyphae that extend at their apices and branch subapically leads to the formation of specialized structures: exploring, secretory, skeletal, storage hyphae, linear or spherical aggregations, etc. The process of differentiation of mycelium occurs unevenly and results in the heterogeneity of the colony. During the last three decades, a number of studies have demonstrated different levels of heterogeneity in solid or even liquid fungal cultures, particularly between microcolonies known as pellets in liquid culture, peripheral and central zones of the colony in surface culture, neighboring hyphae within a zone and even between compartments of a single hypha (for review see [1,2]). Fungal mycelium is heterogeneous in growth, gene expression, biosynthesis, and secretion of proteins and metabolites [3,4,5,6]. For instance, the most active hyphal growth and protein secretion (expression of genes encoding chitin synthase chsF, glucoamylase glaA, and secretion machinery including cnx1, rab6, cypB, hacA) is observed at the periphery of *Aspergillus niger* colony, whereas expression of hydrophobin genes hfbA, hfbB, hfbD is almost exclusively found in the center [5].

The heterogeneity of transcriptome, proteome, and metabolome of the fungal colony is caused mainly by the differences in nutritional and micro-environmental conditions [4,7]. Cell differentiation and aging processes, septal plugging of apical hyphal segments by Woronin bodies as well as oscillations of signaling molecules are considered to be possible mechanisms determining hyphal and zonal heterogeneity [1,2,4]. A significant gradient in the content of metabolites in different zones of the colony can exist despite the fact that the connected hyphae enable cytoplasmic streaming, facilitating the exchange of nutrients between parts of the colony [8]. Some attempts are made to evaluate the spatial distribution of metabolites in fungal and microbial colonies using the MALDI-TOF mass spectrometry imaging methods [9,10,11,12]. These studies allow visualizing the presence of metabolic heterogeneity.

Although the biochemical and molecular mechanisms of mycelium heterogeneity have been the subject of many studies, the role of lipids in colony development and zonality is still not understood. At the same time, recent studies have shown that there is a relationship between fungal growth and development and lipid metabolism. It is known that lipid rafts can participate in the polar growth of hyphal apices [13]; the ratio of major membrane lipids, particularly phosphatidylcholines (PC) and phosphatidylethanolamines (PE), correlates with the age of the fungal colony; the increase in phospholipids esterified by C18:2 Δ9,12 and C18:3 Δ9,12,15 fatty acids accompanies the fruit body development [14]. In *Aspergillus nidulans,* PC affects hyphal extension, membrane integrity, and chitin synthesis [15,16,17]. In a few plant and animal fungal pathogenic fungi, mutants deficient in PC formation have decreased virulence on their hosts [18,19,20,21], but PE biosynthesis mutants are hypervirulent [22].

The progress in lipidomics, which gives information about molecular species of individual lipid classes, initiated a new surge of interest in phospholipid functions. In our previous work based on the results of ESI-MS analyses of PC profiles of 38 fungal species belonging to basidiomycetes, the diversity of PC (20–38 molecular species in every profile) has been demonstrated [23]. This work was undertaken to extend our knowledge of mycelium heterogeneity and to answer the question of how different molecular species are distributed in the surface colony of the basidial fungus *Flammulina velutipes* and how this distribution correlates with its morphology. Considering that the synthesis of phospholipids in fungi occurs in different ways, we also attempted to find out which metabolic pathways are associated with certain molecular species of phospholipids and whether the heterogeneous distribution is a consequence of differential expression and synthesis.

## 2. Materials and Methods

### 2.1. Fungal Strain and Growth Conditions

The strain of *F. velutipes* (Curt.: Fr.) Sing. LE-BIN 1483 used in this study was obtained from the Basidiomycetes Culture Collection of the Komarov Botanical Institute RAS (LE-BIN) (St. Petersburg, Russia). The strain was taxonomically verified by PCR amplification of the 18S-ITS1-5.8S-ITS2-28S region (GenBank accession number KY352493) as described previously [24].

**Figure 1:**
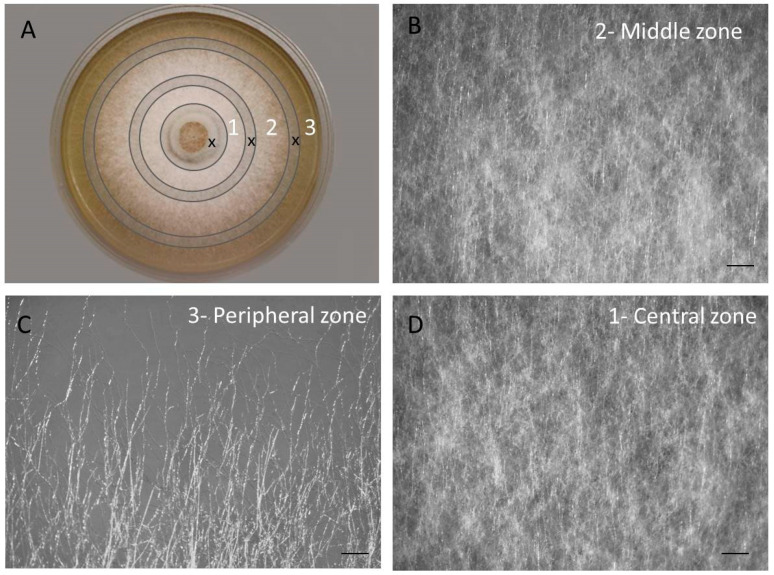
Morphology of the *F. velutipes* colony. (**A**)—Three concentric zones distinguished in the surface colony of *F. velutipes*. The crossed-out areas (x) were removed to avoid overlapping zones. (**B**–**D**)—Hyphae morphology of the colony zones. Scale bar represents 500 µm.

The strain was grown in the dark on the agarized beer wort medium (4% beer, Severnaya Pivovarnya, Saint-Petersburg, Russia; 2% agar Difco, Tucker, GA, USA) at 25 °C. Inoculum plugs (7 mm in diameter) were placed mycelium-side down in the center of Petri dishes (90 mm). Cultures were harvested after 7 days of growth when the mycelial colony reaches the edge of the Petri dish. A total of 3 zones of colony were determined based on visual observations under light stereomicroscope Zeiss Stemi 2000 (Jena, Germany) (Figure 1).

### 2.2. Labeling Experiments

Seven-day colonies of *F. velutipes* were sprayed with the water solutions of d9-choline (Sigma-Aldrich, Darmstadt, Germany), d4-ethanolamine (Sigma-Aldrich, Darmstadt, Germany), or d9-choline + d4-ethanolamine at the final concentration of each phospholipid precursor 3 mM, incubated during 24 h and then carefully scrapped from agar surface into isopropanol for lipid extraction. Control colonies were sprayed with water.

### 2.3. Lipid Extraction

The extraction of lipids from fresh mycelium was performed by isopropanol and isopropanol–chloroform (1:1), according to Nichols et al. [25]. For each replicate, the mycelia of 10 colonies were combined. The fresh mycelia of each zone were sampled by scrapping off with a scalpel blade, homogenized in a mortar with isopropanol, and heat treated at 70 °C for 30 min. After cooling to room temperature, the homogenate was centrifuged (4000× *g*, 10 min), followed by the transfer of supernatant to a fresh tube. The pellet was resuspended in isopropanol–chloroform (1:1), then extracted a second time, as mentioned above, and centrifuged. The combined extracts were evaporated, dissolved in chloroform–methanol (1:1), and washed clean of water-soluble impurities with a 2.5% NaCl solution. The obtained extract was evaporated and dissolved in a mixture of chloroform–methanol (1:1), then stored at −20 °C.

### 2.4. Separation of Phospholipid Classes by TLC 

The content of lipid classes was determined densitometrically after the separation using two-dimensional TLC according to Bening et al. [26] on silica gel 60 plates 10 × 10 cm (Merck, Darmstadt, Germany). The typical TLC chromatogram is depicted in Figure S1. The amounts of lipid classes were determined using a Denscan densitometer (Lenchrom, Saint-Petersburg, Russia) after visualization by heating at 140 °C with a 5% sulfuric acid solution in methanol. Phosphatidylcholine (PC) from egg yolk and bovine cerebrosides used as standards on TLC were obtained from Sigma-Aldrich (Darmstadt, Germany). The zones corresponding to major phospholipids (PC and PE) were scraped off the plate, followed by elution with chloroform–methanol (1:1) for MS/MS analysis of molecular species composition.

### 2.5. LC-MS/MS Analysis 

The resulting PC and PE fractions were analyzed using the Shimadzu LCMS-9030 QqTOF mass spectrometer coupled with the Nexera X2 UPLC system. Luna Omega C18 column (2.1 × 100 mm, 1.6 µm, 100 Å) with SecurityGuard guard column was used; injection volume was 0.5 μL (positive). Binary gradient: 45%B (0 min)–100%B (11 min)–100%B (13 min)–45%B (14 min)–45%B 14.1 min (stop time). The flow rate was 0.4 mL/min, and the column oven temperature was set at 65 °C. Mobile phases as follows: water/methanol (1:1) as solvent A and 95% 2-propanol as solvent B, both containing ammonium formate (10 mM), H3PO4 (8 µM), and formic acid (0.1 vol%) (as described in Knittelfelder et al. [27]). The example of the total ion chromatogram and extracted ion chromatogram of phosphatidylcholine fraction is depicted in Figure S1A,B, respectively. Blank control and three quality control (QC) samples consisting of a mixture of all biological samples were regularly interspersed to ensure the quality of the batch.

The high-resolution mass spectra were recorded in the positive ion mode. The data-independent Sequential Window Acquisition of All Theoretical Mass Spectra (SWATH-MS) method, where MS/MS spectra were acquired for all detectable lipid species, was used [28]. The parameters were set as follows: MS1 mass range, 640–1050 m/z, event time 100 ms; MS2 mass range, 150–1050 m/z, event time 30 ms; collision energy, 30 V; collision energy spread, 5 V; Q1 resolution, 14.1 m/z; loop time 970 ms. The following ESI parameters were used: interface heater temperature, 180 °C; heating gas flow, 10.0 L/min, nebulizing gas flow 3.0 L/min, dry gas flow 10.0 L/min, DL temperature 250 °C, heat block temperature 250 °C interface voltage, 4500 V (+), 3500 V (−) (see parameters in Table S1).

Processing of the SWATH-MS dataset (detection of peaks, MS2 data deconvolution, compound identification, and alignment of peaks through all the samples) was performed in MS-DIAL software (RIKEN, version 4.60) [29]. Lipid identification was checked manually to remove false positive identifications based on the MS/MS fragments (see Table S2). The example of a spot plot (each spot denotes the detected precursor ion) after processing SWATH data by MS-DIAL is depicted in Figure S2C. 

An equimolar mixture of 13:0/13:0 PC, 15:0/15:0 PE, and 19:0/19:0 PC molecular species (Avanti Polar Lipids, Alabaster, Montgomery, AL, USA) indicated that the signal intensities reflect the relative abundance of the PC molecular species under the conditions used.

For the targeted search of deuterium-labeled PC species, precursor ion scan mass spectrometry was conducted using LCMS-8030 QqQ instrument with collision energy −34 V. chromatographic conditions were the same as for SWATH analysis.

### 2.6. Gene Orthology Search 

A gene orthology search was performed using the BLAST program [30] on the NCBI website for all of the databases available as of 1 September 2022 and at the http://web.seeders.co.kr/fve/index.php/fve/blast (accessed on 1 February 2022). Sequences were aligned using the ClustalW interface of the Mega 6.06 software [31]. Identification of conserved domains was performed using CD—search service in CDD database version 3.19 [32].

### 2.7. RNA Isolation

For RNA extraction, mycelium was ground from the agarized medium with a scalpel (for each of 3 replicates, the mycelium of 10 colonies were pooled), then mycelium was ground to a powder in liquid nitrogen and stored at −80 °C. Total RNA was isolated using the RNeasy Plant Mini kit (Qiagen, Valencia, CA, USA) according to the instructions of the manufacturer. The CleanRNA Standard kit was used to clean up the total RNA (Eurogen, Moscow, Russia).

The integrity of the extracted total RNA samples was verified via electrophoresis on a 1.4% agarose gel followed by ethidium bromide staining. Purity and concentration were determined using a spectrophotometer (NanoDrop Lite, Thermo Scientific, Wilmington, CA, USA) and fluorometer (Qubit™ fluorometer apparatus, Invitrogen, Carlsbad, CA, USA).

### 2.8. Quantitative Real-Time Reverse Transcriptase Polymerase Chain Reaction (qRT-PCR)

cDNA templates for all quantitative real-time reverse transcriptase polymerase chain reactions (qRT-PCR) were synthesized from 1000 ng of total RNA using the MMLV RT kit (Evrogen, Moscow, Russia) following the manufacturer’s protocol. All gene-specific primers (Table 1) were designed using the online resource Primer-BLAST at https://www.ncbi.nlm.nih.gov/tools/primer-blast (accessed on 1 February 2022) [33] or Beacon Designer software and synthesized from Evrogen, Russia. The qRT-PCR experiments were performed on an Eco Real-Time PCR System (Illumina, Inc., San Diego, CA, USA). Amplification efficiency for each primer set was calculated by serially diluting the exponential-phase cDNA template. 

Melting curve analysis was performed for each pair of primers after each run in an Eco Real-Time PCR System instrument to confirm the specificity of the primers. 

DNA contamination was checked by (no reverse transcription) PCR for each RNA sample in Eco Real-Time PCR System (Illumina, Inc., San Diego, CA, USA) using target primers. No amplification was seen after 35 cycles of amplification.

The specificity of each primer pair was verified via electrophoresis on a 1.4% agarose gel followed by ethidium bromide staining. The PCR products were directly purified from the reaction mixture using the Cleanup S-Cap kit (Evrogen, Russia) and sequenced using the Sanger method. Only one band was observed in the gel.

All qRT-PCR experiments were performed on an Eco Real-Time PCR System (Illumina, Inc., USA). The amplification reaction was carried out using a ready-made mixture of PCR qPCRmix-HS SYBR + ROX (Evrogen, Russia) according to the manufacturer’s instructions. A total of 50 ng of cDNA and gene-specific primers (Table 1) at concentrations of 0.2–0.4 µM were added to the reaction mixture with a volume of 20 µL (the concentration was optimized for each pair). Amplification program: 5 min at 95 °C, 45 cycles (95 °C 15 s, 58 °C 20 s, 72 °C 30 s), and melting reaction product (Melting Curve program: 70–95 °C, +1 °C/s).

The mRNA level was normalized to the expression of 18S rRNA, which was constantly expressed under all experimental conditions. It was chosen from 4 gene candidates: 18S rRNA, actin, GAPDH, and VP26 using BestKeeper software [34]. The qRT-PCR results were elaborated using the 2^−ΔCT^ method [35].

### 2.9. Statistics

All experiments were carried out in 3–4 replicates. Four independent experiments were conducted; the paper presents the results of the most representative experiment. Statistical analyses were performed using Microsoft Excel software. Statistical significance between experimental groups was determined using the Student’s *t*-test (confidence interval 95%) [36].

## 3. Results

### 3.1. The Colony of F. velutipes Is Morphologically Heterogenous

Three concentric zones were distinguished in the surface colony of *F. velutipes* (Figure 1A). The peripheral zone of the colony (1 cm from the edge) consisted of actively growing weakly branching or unbranching, undifferentiated hyphae (Figure 1C). In the middle zone of the colony, the hyphae were branched and thickly intertwined; mycelium was uniform, without aggregations and differentiation. The central zone of the colony, with a diameter of 2 cm, consisted of a dense mycelium, over the entire thickness of which numerous spherical aggregations of hyphae were observed.

### 3.2. Lipid Composition Demonstrated Distinct Patterns across the F. velutipes Colony

**Figure 2:**
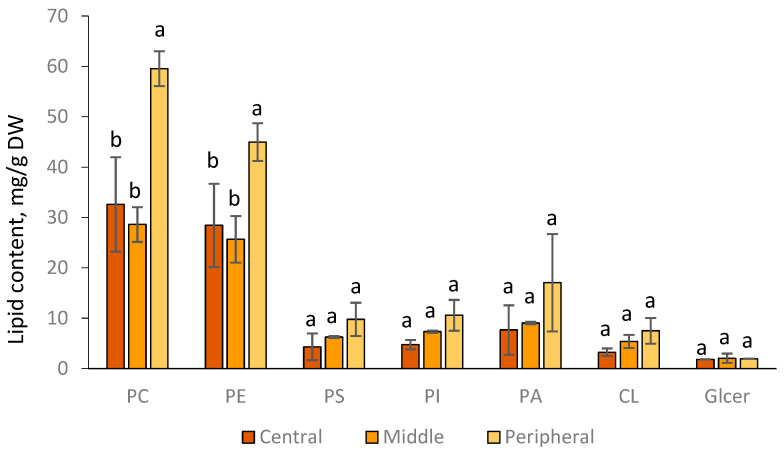
The composition of lipid classes in different zones of the *F. velutipes* colony. The experiments were performed in triplicate, and error bars indicate standard deviations. Different letters above the bars indicate statistically significant differences at *p*-value < 0.05 (*t*-test). PC –phosphatidylcholine; PE—phosphatidylethanolamine; PS—phosphatidylserine; PI—phosphatidylinositol; PA—phosphatidic acid; CL—cardiolipin; GlCer—glycoceramide.

The content of phospholipid classes varied in different zones of the *F. velutipes* colony (Figure 2). The peripheral zone of the colony was rich in phospholipids (1.8 times more than in the middle and central zones) and characterized by a higher value of the phosphatidylcholine (PC) to phosphatidylethanolamine (PE) ratio compared with the central zone of mycelium. 

**Figure 3:**
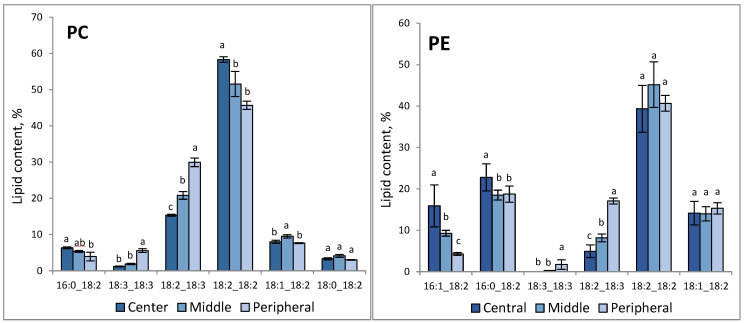
The content of major phospholipid species in different zones of *F. velutipes* colonies. The experiments were performed in triplicate, and error bars indicate standard deviations. Different letters above the bars indicate statistically significant differences at *p*-value < 0.05 (*t*-test).

The composition of molecular species of PC and PE was performed by LC-MS mass spectrometry after the preliminary separation of the lipid classes using TLC. Figure 3 represents major molecular species (whose content was higher than 5% of the sum of all molecular species of a given class in at least one of the zones). A full list of molecular species is in Table S3. PC was represented by 29 molecular species, PE—14 species. In the peripheral zone of the colony, PC and PE were enriched in the molecular species containing linolenic acid. In particular, at the colony periphery, 18:2_18:3 PC were 27% of PC, which is 1.4 times more than in the middle zone and two times more than in the center. Di-linolenate PC (18:3_18:3 PC) accumulated in the peripheral zone up to 5%, while in the central zone, 18:3_18:3 PC amounted to only 1%. A similar trend was observed in PE where 18:2_18:3 PE reached 17% in the peripheral zone as compared with 5% in the center. In the central zone of the colony, an increase in the relative content of 16/18 phospholipids, compared to the peripheral zone, was registered: 16:0_18:2 PC increased from 3.5% to 6%, 16:0_18:2 PE—from 19% to 23%, and 16:1_18:2 PE—from 4% to 16%.

### 3.3. Identification of Genes Participating in PE and PC Biosynthesis

Since changes in the molecular composition of lipids are thought to be driven by the regulation of gene expression, in the next step, we analyzed the mRNA abundance of lipid biosynthetic genes.

**Figure 4:**
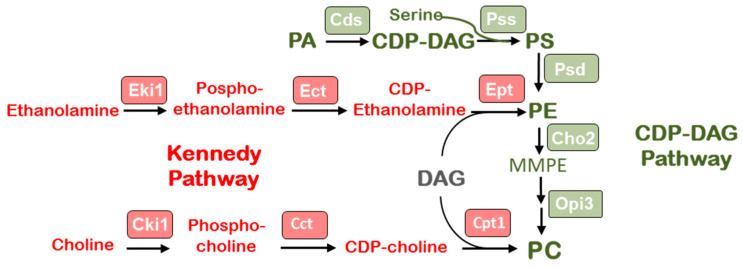
The scheme of phospholipid metabolism in fungi.

**Figure 5:**
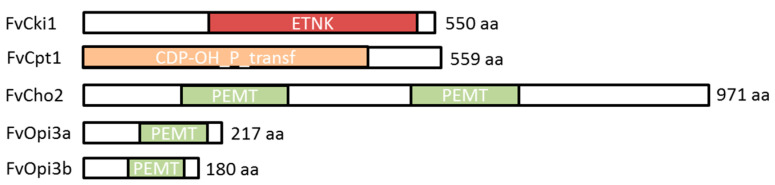
Predicted conserved domains in the phospholipid biosynthetic enzymes of *F. velutipes*. CDP-OH_P_transf—CDP-alcohol phosphatidyltransferase (cl00453); ETNK—eukaryotic ethanolamine kinase (cd05157); PEMT—phospholipid methyltransferase (pfam04191).

In yeast cells, PC is synthesized in two ways: through the Kennedy pathway or the CDP-DAG pathway (Figure 4) [37,38]. Unfortunately, there is no information in the literature about what pathway phospholipids are synthesized in basidial fungi. To reveal the PC biosynthesis genes in *F. velutipes*, we analyzed genome sequences of *F. velutipes* 4019-20 strain produced by the Korea Rural Development Administration http://112.220.192.2/mushroom/ (accessed on 9 January 2022) and draft genome sequences of *F. velutipes* KACC42780 strain deposited at GenBank (http://www.ncbi.nlm.nih.gov/ (accessed on 9 January 2022)) under the accession number AQHU00000000 [39]. The sequences of the enzymes involved in the PE and PC biosynthetic pathways in yeast *Saccharomyces cerevisiae* and recently characterized enzymes of mycelial fungus *F. graminearum* [40] were used as queries for BlastP searches to identify homologous proteins of *F. velutipes* (Table 2). The identified proteins shared relatively low identity (31–54%) with their homologs, but structural analysis revealed that these proteins contained the same domain architecture (Figure 5).

BLAST searches suggested that *F. velutipes* chr08_NT_00720 is homologous to both yeast choline kinase (Cki1) and ethanolamine kinase (Eki1), suggesting that the protein encoded by the chr08_NT_00720 locus may have both choline kinase and ethanolamine kinase activities. The identity of this protein with yeast bifunctional choline kinase/ethanolamine kinase Cki1 (NP_013234.1) is 31.5%. We designated it as FvCki1.

**Figure 6:**
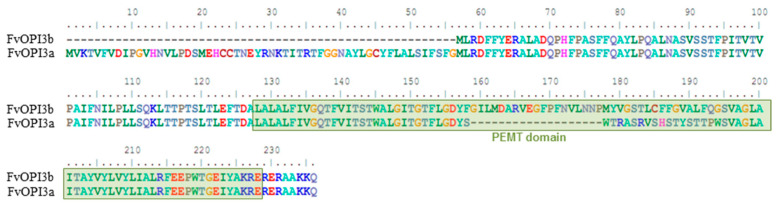
A comparison of the amino acid sequence of the two isoforms of *F. velutipes* Opi3 proteins. The PEMT domain is circled in green.

It was shown that the *F. velutipes* genome contains two separate OPI3 genes coding class I phospholipid methyltransferase (PLMT) in the *F. velutipes* genome—in 4 and 10 chromosomes. In Figure 6, the deduced amino acid sequences of two Opi3 isoforms are aligned so that maximum matching is obtained. The protein encoded by the gene on the 10 chromosome (designed as OPI3a) has 217 amino acids, whereas the protein encoded by the gene on the 4 chromosome (OPI3b) has 180 amino acids. Opi3a has 19 amino acid deletions within the PEMT domain region.

### 3.4. Genes of Lipid Metabolism Are Differentially Expressed across the F. velutipes Colony

**Figure 7:**
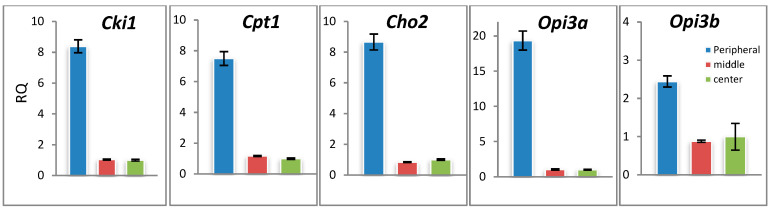
Zonal heterogeneity of gene expression in *F. velutipes* colony. RQ—relative transcript quantities. The experiments were performed in triplicate, and the data are presented as means ± SD.

The expression of two genes of the Kennedy pathway (CKI1 and CPT1) and three genes of the CDP-DAG pathway (CHO2, OPIa, and OPI3b) was measured. The real-time PCR analysis showed that the RNA abundance of all studied genes was higher in the peripheral zone of *F. velutipes* colonies (Figure 7). However, the elevation of transcript abundance in the peripheral zone of the colony was not the same for different genes: the largest fold change was observed for the Opi3a gene, which may indicate an increase in the CDP-DAG pathway in the periphery hyphae.

### 3.5. Biosynthesis of PC Molecular Species Occurs Predominantly via Kennedy Pathway

To distinguish the PC species synthesized from the Kennedy pathway and the CDP-DAG pathway in *F. velutipes* and answer the question does the biosynthesis of PC molecular species differ in different zones of a colony, we conduct experiments with deuterium-labeled phospholipid precursors uptake. For this, 7-day colonies of *F. velutipes* were sprayed with the water solution containing labeled *d9*-choline, *d3*-serine, and *d4*-ethanolamine, incubated for 24 h and after that, carefully scraped from the agar surface for lipid extraction.

**Figure 8:**
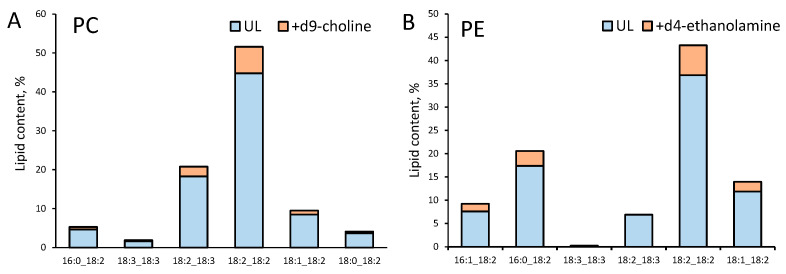
Incorporation of *d9*-choline in PC (**A**) and *d4*-ethanolamine in PE (**B**) molecular species in the middle zone of *F. velutipes* colony. Blue bars—unlabeled (UL) molecular species, orange bars—*d9*-choline- or *d4*-ethanolamine-labeled species.

The incorporation of labeled precursors in PC and PE was registered by SWATH mass spectrometry. In positive ion mode, MS/MS spectra of non-labeled PC molecules have a characteristic fragment of m/z 184 corresponding to the protonated phosphocholine head group. PC species synthesized by the Kennedy pathway incorporated labeled *d9*-choline in their head group and had a characteristic fragment of m/z 193 in the MS/MS spectra. Similarly, molecular species of PC derived from PE methylation (CDP-DAG pathway) incorporated labeled *d4*-ethanolamine and had a polar fragment of m/z 188 in their MS/MS spectra. It was shown that about 10% of each molecular species of PC incorporated labeled *d9*-choline (Figure 8A), whereas the incorporation of *d4*-ethanolamine was not registered. 

**Figure 9:**
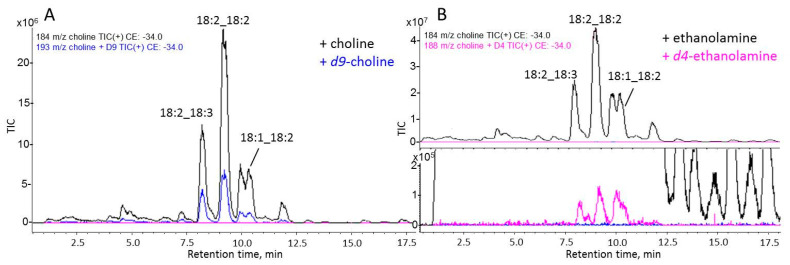
LC-MS/MS precursor ion scan (TIC) chromatogram. Data observed from the targeted analysis of *d9*-choline (**A**) and *d4*-ethanolamine (**B**) incorporation in PC.

To identify *d4*-ethanolamine-labeled PC species, we had to use the method of target mass spectrometry—precursor ion scan. Figure 9 depicts the results of the precursor ion scan of m/z 184, 188, and 193 of PC extract of *F. velutipes*. The three small peaks depicted in Figure 9B confirm the presence of trace amounts of *d4*-ethanolamine-labeled PC. 

Taken together, these results suggest that the Kennedy pathway is the predominant route for PC biosynthesis in *F. velutipes*.

PE molecular species were detected by the loss of a neutral fragment of m/z 141, corresponding to the phosphoethanolamine head group. Mass spectra of d*4*-ethanolamine-labeled PE included fragments corresponding to the neutral loss of 145. It was shown that PE consisted of 10% of *d4*-labeled molecular species (Figure 8B). *D3*-serine-labeled PE species were not registered. 

## 4. Discussion

The development of our knowledge of the chemistry and biology of mycelial fungi at the macro- and micro-levels from a colony to a single hypha demonstrates that processes such as transcription, translation, and metabolism are different in space across cells. Multiscale heterogeneity within the area of fungal colony involves lipid metabolism, which can be regulated externally by the supply of precursor lipids, acetyl-CoA as well as the local availability of dissolved oxygen and light [41] or internally through genes and enzymes of phospholipid biosynthesis, the gradient of signal molecules and lipid transporters, cytoskeleton reorganization [42,43,44].

According to the results obtained, the expression of genes of PC metabolism, accumulation of phospholipid classes including PC, PE, PS, PI, PA, CL, and degree of unsaturation of PC and PE increased in the direction from the center to the periphery of the colony. The peripheral zone of the colony was characterized by a higher value of the PC/PE ratio, which confirms our previous hypothesis on the existence of a physiologically determined relationship between the relative amount of PC and the hyphae differentiation. We have previously demonstrated that young 7-day vegetative mycelium of *F. velutipes* contained the largest relative amount of PC, which was later exhausted during the development of culture [14].

Lipidomic analysis revealed significant differences in the major molecular species of PC and PE between the central and peripheral zones of the colony (Figure 3, Supplemental Table S3). The peripheral zone differed in the high value of 18:2_18:3 and of 18:3_18:3 species of PC and PE, whereas phospholipids of the central zone were enriched in 16:0_18:2 and 16:1_18:2 species of PC and PE. Carbon and nitrogen limitation, as well as phosphorus deficiency in the medium of the central zone, are the main factors influencing phospholipid production [41]. However, the regulation by means of nutrient supply seems to be not effective as regards α-linolenic acid accumulation.

Previously it was reported that the development of the surface culture of *F. velutipes* was accompanied by changes in the molecular composition of phospholipids, with linolenic acid accumulating in PC and PE at the early stage of development when polar growing non-differentiated hyphae dominated [14]. The increase in the content of C18:3 acid in phospholipids of actively growing non-differentiated mycelium can be due to their involvement in the synthesis of oxylipins possessing hormonal activity. For example, in an ascomycete *A. nidulans,* oxygenated derivatives of linoleic acid, 8-hydroxy-9,12-octadecadiene, and 8,11-dihy-droxy-9,12-octadecadiene acids were demonstrated to induce the formation of ascospores and to inhibit conidia formation [45].

Another reason for the accumulation of C18:3-containing phospholipids in the growing edge of the colony is its possible participation in lipid-protein interactions. Last year, there were new reports of the participation of individual lipid species in the topological organization of proteins [46]. Several lipids, including PUFAs, have been suggested to play modulatory roles in the activation process and permeation across various K^+^ selective channels by binding to lipid-specific pockets [47].

The peripheral zone of the colony is enriched in vegetative, actively growing hyphae. Therefore, it can be assumed that the accumulation of linolenic acid in this zone is associated with its participation in the processes of polar growth. Polarized growth of hyphae requires a sequential supply of proteins and lipids to the hyphal tip. There are many reports devoted to the role of sterol-rich lipid rafts in fungal polar growth [13,48,49]. Recent work demonstrated that lipid raft organization is uniquely modified by n-3 polyunsaturated fatty acids [50]. The specific mechanism of action of linolenic acid in the growing hyphae of fungi will be the subject of further studies.

The analysis of identified genes made it possible to reconstruct the preliminary phospholipid metabolism scheme of *F. velutipes*. It is known that in yeast, PC is synthesized by two pathways: predominantly by the CDP-DAG pathway (also known as methylation pathway or de novo biosynthesis) and through CDP-choline (Kennedy pathway) [36]. In the *F. velutipes* genome, we identified enzymes of both metabolic pathways. However, unlike yeast, in which the phosphorylation of choline and ethanolamine (the first step of Kennedy’s pathway) is carried out by two related enzymes—choline kinase CKI and ethanolamine kinase EKI1, in the *F. velutipes* genome, we were able to identify only one gene containing the ETNK_EUK functional domain, characteristic for cholineand ethanolamine kinases. A similar thing is observed in *F. graminearum*, the genome of which encodes only one gene of choline/ethanolamine kinase FgCki1 [40]. Since yeast kinases CKI и EKI1 were shown to have both activities and differ only in the degree of substrate specificity to choline and ethanolamine [51], we can suggest that the kinase identified in *F. velutipes* is also bifunctional and involved in the biosynthesis of PC and PE. Another feature of *F. velutipes* compared to yeast is that PLMT exists in *F. velutipes* as two isoforms encoded by two separate genes named *OPI3a* and *OPI3b*. The elevation of Opi3b transcript abundance in the peripheral zone of the colony measured by real-time PCR was much lower than Opi3a. Evidently, these isoforms are functionally different.

The real-time PCR analysis showed that the RNA abundance of two genes of the Kennedy pathway (CKI1 and CPT1) and three genes of the CDP-DAG pathway (CHO2, OPIa, and OPI3b) was higher in the peripheral zone of *F. velutipes* colonies that is in agreement with the previous reports. For example, RNA content per hypha in *Aspergillus niger* microcolonies in liquid shaken cultures was about 45 times higher at the periphery than in the center of the microcolony [52]. However, the elevation of transcript abundance in the peripheral zone of the colony was not the same for different genes: the largest fold change was observed for the Opi3a gene, which may indicate an increase in the CDP-DAG pathway in the periphery hyphae. 

The real-time PCR method allows us to measure the relative intensity of gene expression but is unable to determine absolute mRNA abundance to compare the intensity of expression of different genes with each other since the amplification efficiencies are affected by the efficiency of primers. So, to check the hypothesis that the CDP-DAG pathway increase in the periphery hyphae, we studied the metabolism of PC biosynthesis using labeled precursors. The distinction between PC molecular species synthesized by the Kennedy pathway and the CDP-DAG pathway can be carried out by mass spectrometry using deuterium-labeled phospholipid precursors [53]. Our experiments demonstrated that *d9*-choline incorporation in PC was significantly higher than *d4*-ethanolamine, which suggests the predominance of the Kennedy pathway in the biosynthesis of PC throughout the colony (Figure 8). The primary route of PC biosynthesis in yeast is the CDP-DAG pathway, but the Kennedy pathway intensifies in response to choline supplementation in the culture medium [54]. Unlike yeast, basidial fungi are capable of synthesizing choline. In our experiments, the labeled solution contained serine, choline, and ethanolamine simultaneously. Probably, this additionally stimulated the Kennedy pathway in the fungal mycelium. Even when we conduct additional experiments only with labeled *d4*-ethanolamine, we can register only trace amounts of *d4*-labeled PC (Figure 9). There were no differences in the *d9*-choline and *d4*-ethanolamine incorporation intensity in PC between the central and peripheral zones of the colony as well as between different molecular species.

Thus, the heterogeneity in lipid metabolism and lipid composition of the fungal mycelium was demonstrated. The zonal differences in gene expression and lipid composition can be explained by exhaustion of the medium, polar growth, participation of membrane lipids in the regulation of polar growth and aging processes, and other factors. The heterogenicity of the colony is a very important and intriguing phenomenon that is perhaps at the heart of such processes as hyphal differentiation and mushroom morphogenesis.

The knowledge of the mechanisms of heterogeneity can be used to optimize the yield of the desired lipid metabolite when using fungi as cell factories. On the other hand, the presence of heterogeneity in the colony requires more accurate planning of the experiment without averaging the whole colony.

## Figures and Tables

**Table 1:** Primers used in this study.

Protein	Gene	Name	Sequence 5′→3′
Choline kinase/Ethanolamine kinase	*cki1*	CKI_Flam_Fw	CACTCTCCACATCCTATCTTCC
		CKI_Flam_Rev	CCAGCCATTGCCTTCGC
Cholinephospho-transferase	*cpt1*	CPT1_Flam_Fw	TTCGTGCCGTTCCTCTGC
		CPT1_Flam_Rev	ATACTCCATATCCACATCCAATCC
Phosphatidylethanolamine- methyltransferase	*cho2*	PEMT_Flam_Fw	TGTTCAACCTGTCTCTTCTGG
		PEMT_Flam_Rev	CGTTTCTTGCTCTGCTTGG
Phospholipid methyltransferase	*opi3a* (4 chromosome)	OPI3_Flam-chr_4_Fw	CACGCCAACAAGCCTTAC
		OPI3_Flam-chr_4_Rev	ACGCCGAAGAAGCAGAG
Phospholipid methyltransferase	*opi3b* (10 chromosome)	OPI3_Flam-chr_10_Fw	GCACGAATGAATACCGCAAC
		OPI3_Flam-chr_10_Rev	AGAACGACGCAGGGAAATG
18S rRNA	18S rRNA	18S_rRNA _Flam_Fw	TGATGTGTTGTTCGGCAC
		18S_rRNA_Flam_Rev	AGTTATGTCTGGACCTGG

**Table 2:** Predicted proteins involved in the phospholipid biosynthesis pathways in *F. velutipes*.

Protein in *S. cerevisiae*	Protein in *F. graminearum*	Function	Homologs in *F. velutipes*	Identities (%)	E-Value
Cki1/Eki1 (NP_013234.1)	FgCki1 (FG05_09539)	Choline kinase/Ethanolamine kinase	FvCki1 (chr08_NT_00720)	42.3 (F.g. ^a^) 31.1 (S.c. ^b^)	4 × 10^−26^ (F.g.) 1 × 10^−52^ (S.c.)
Cpt1 (AJU22210.1)	FgCpt1 (FGSG_09402)	Cholinephosphotransferase	FvCpt1 (chr07_AA_00140)	33.2 (F.g.) 34.0 (S.c.)	5 × 10^−67^ (F.g.) 1 × 10^−74^ (S.c.)
Cho2 (NP_011673.1)	FgCho2 (FGSG_05066)	Phosphatidylethanolamine N-methyltransferase (II class PLMT)	FvCho2 (chr04_AA_00051)	35.0 (F.g.) 40.8 (S.c.)	0.0 (F.g.) 6 × 10^−65^ (S.c.)
Opi3 (AJR72524.1)	FgOpi3 (FGSG_08613)	Phospholipid methyltransferase (I class PLMT)	FvOpi3a (chr10_NT_00876)	37.0 (F.g.) 31.1 (S.c.)	5 × 10^−39^ (F.g.) 2 × 10^−27^ (S.c.)
			FvOpi3b (chr04_NT_00638)	55.9 (F.g.) 57.6 (S.c.)	5 × 10^−48^ (F.g.) 3 × 10^−47^ (S.c.)

Note. ^a,b^ The protein identity and E-value of *F. velutipes* sequences are shown in relation to *Fusarium graminearum* (F.g.) and *Saccharomyces cerevisiae* (S.c.) counterparts.

## Data Availability

Data are contained within the article.

## Supplementary Materials

The following supporting information can be downloaded at: https://www.mdpi.com/article/10.3390/jof9010102/s1, Figure S1: The typical TLC chromatogram of *F. velutipes* lipid extract; Figure S2: The example of TIC, EIC chromatograms, and SWATH plot; Table S1: SWATH settings; Table S2: PC and PE species of *F. velutipes* identified by SWATH-MS; Table S3: The content of phospholipid species in different zones of *F. velutipes* colony.

## Abbreviations

CKI—choline kinase; CL—cardiolipin, CPT—cholinephosphotransferase; DAG—diacylglycerol; EKI—ethanolamine kinase; ESI-MS—electrospray mass spectrometry; GlCer—glycoceramide; PA—phosphatidic acid; PC—phosphatidylcholine; PE—phosphatidylethanolamine; PEMT—phospholipid methyltransferase; PI—phosphatidylinositol; PS—phosphatidylserine; SWATH—Sequential Window Acquisition of All Theoretical Mass Spectra; TLC—thin-layer chromatography.
