# Supplementary material for: Heterogeneous Distribution of Phospholipid Molecular Species in the Surface Culture of Flammulina velutipes: New Facts about Lipids Containing α-Linolenic Fatty Acid
Source: J Fungi (Basel). 2023 Jan 12;9(1):102. doi: 10.3390/jof9010102 (PMC9865325; doi:10.3390/jof9010102)
Supplement: Supplementary file 1 [file jof-09-00102-s001.zip › Figure S1. TLC.pdf]

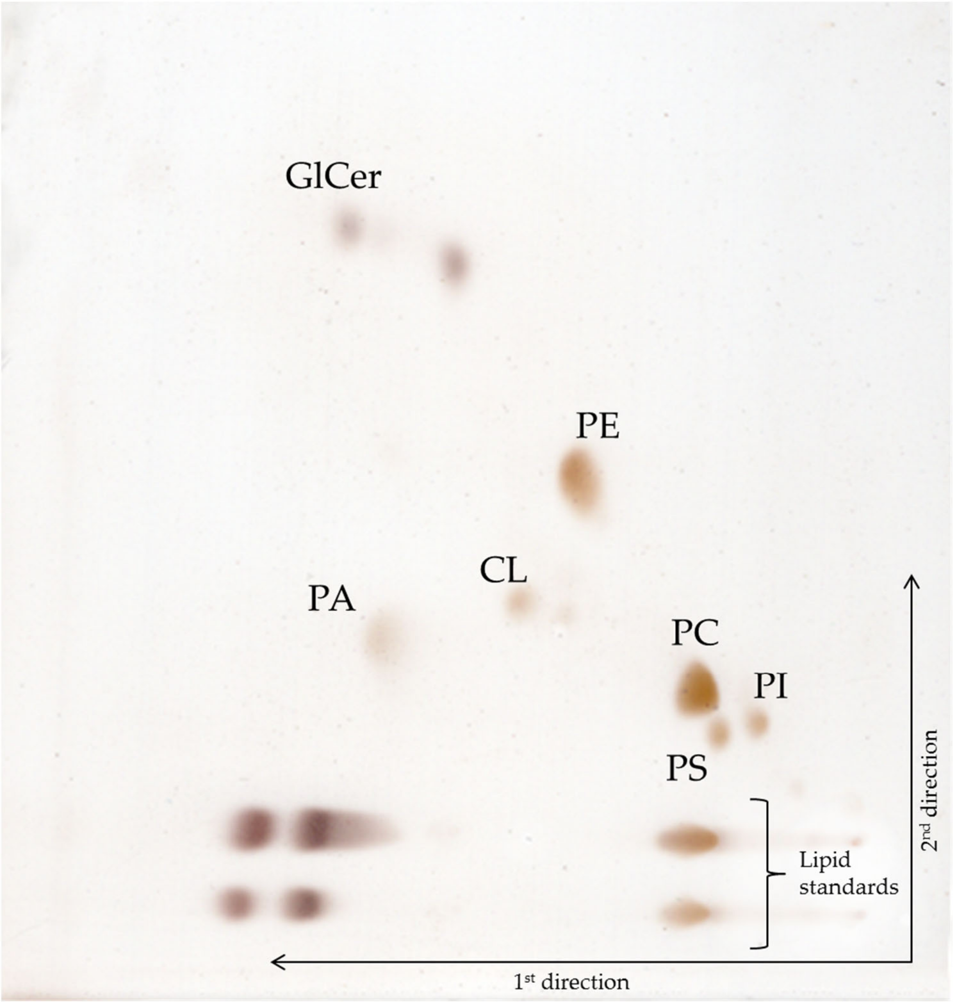

Figure S1. The typical TLC chromatogram of *F. velutipes* lipid extract obtained with two-dimensional TLC according to Bening et al. (1995) on silica gel 60 plates 10 × 10 cm (Merck, Germany).
