# Supplementary material for: Heterogeneous Distribution of Phospholipid Molecular Species in the Surface Culture of Flammulina velutipes: New Facts about Lipids Containing α-Linolenic Fatty Acid
Source: J Fungi (Basel). 2023 Jan 12;9(1):102. doi: 10.3390/jof9010102 (PMC9865325; doi:10.3390/jof9010102)

A. The example of total ion chromatogram of *F. velutipes* phosphatidylcholine fraction

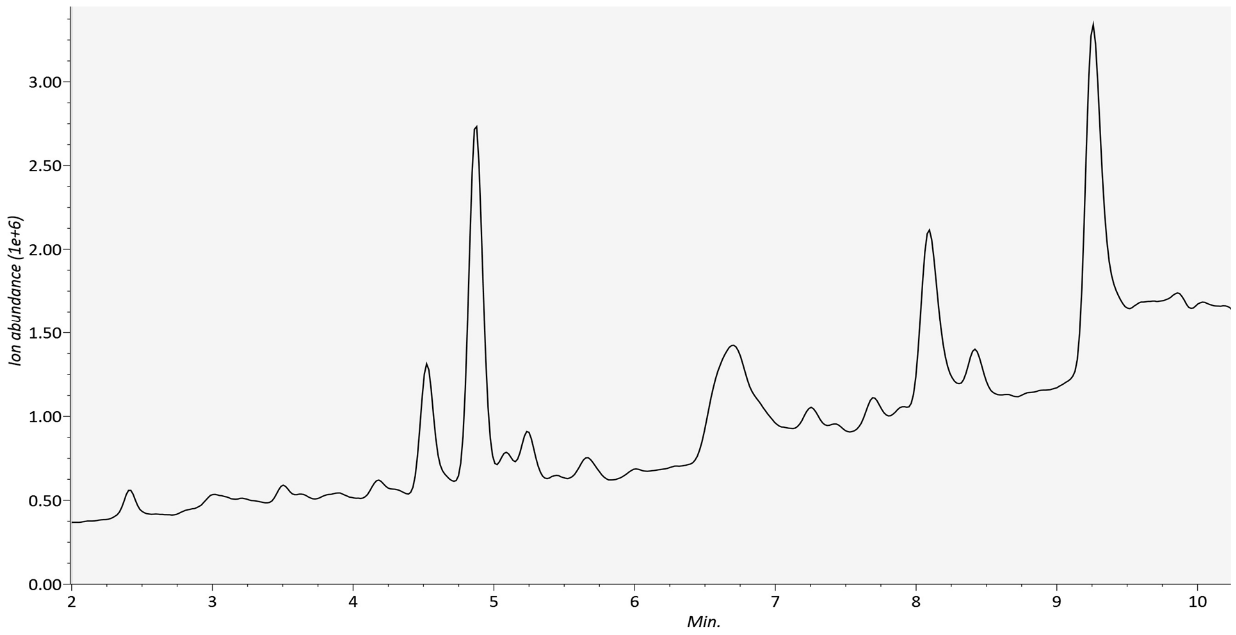

B. The example of extracted ion chromatogram of *F. velutipes* phosphatidylcholine fraction

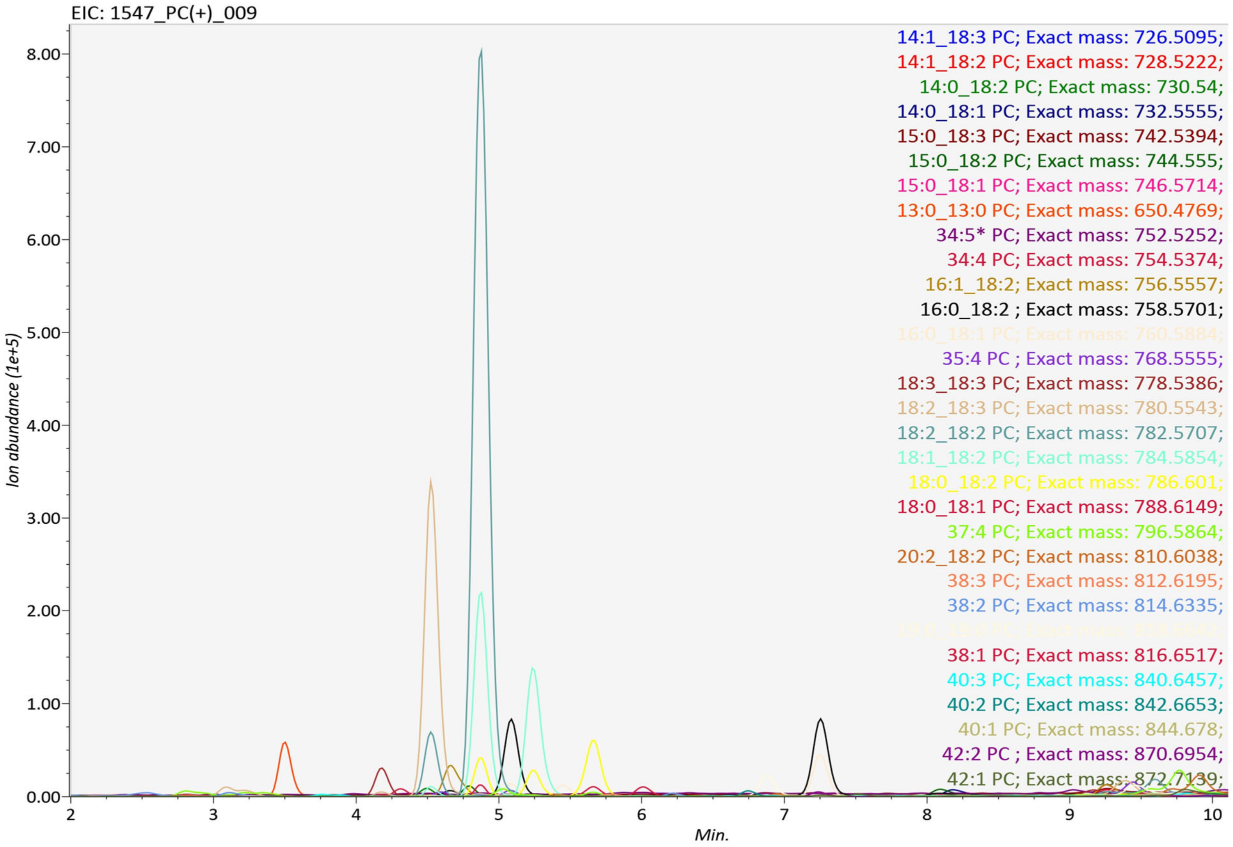

EIC: 1547_PC(+)_009
14:1_18:3 PC; Exact mass: 726.5095;
14:1_18:2 PC; Exact mass: 728.5222;
14:0_18:2 PC; Exact mass: 730.54;
14:0_18:1 PC; Exact mass: 732.5555;
15:0_18:3 PC; Exact mass: 742.5394;
15:0_18:2 PC; Exact mass: 744.555;
15:0_18:1 PC; Exact mass: 746.5714;
13:0_13:0 PC; Exact mass: 650.4769;
34:5* PC; Exact mass: 752.5252;
34:4 PC; Exact mass: 754.5374;
16:1_18:2; Exact mass: 756.5557;
16:0_18:2 ; Exact mass: 758.5701;
16:0_18:1 PC; Exact mass: 760.5884;
35:4 PC ; Exact mass: 768.5555;
18:3_18:3 PC; Exact mass: 778.5386;
18:2_18:3 PC; Exact mass: 780.5543;
18:2_18:2 PC; Exact mass: 782.5707;
18:1_18:2 PC; Exact mass: 784.5854;
18:0_18:2 PC; Exact mass: 786.601;
18:0_18:1 PC; Exact mass: 788.6149;
37:4 PC; Exact mass: 796.5864;
20:2_18:2 PC; Exact mass: 810.6038;
38:3 PC; Exact mass: 812.6195;
38:2 PC; Exact mass: 814.6335;
19:0_19:0 PC; Exact mass: 818.6642;
38:1 PC; Exact mass: 816.6517;
40:3 PC; Exact mass: 840.6457;
40:2 PC; Exact mass: 842.6653;
40:1 PC; Exact mass: 844.678;
42:2 PC ; Exact mass: 870.6954;
42:1 PC; Exact mass: 872.7139;
Ion abundance (1e+5)
8.00
7.00
6.00
5.00
4.00
3.00
2.00
1.00
0.00
2
3
4
5
6
7
8
9
10
Min.

C. The example of spot plot of phosphatidylcholine molecular species after processing of SWATH data by MS-DIAL software (RIKEN, version 4.60)

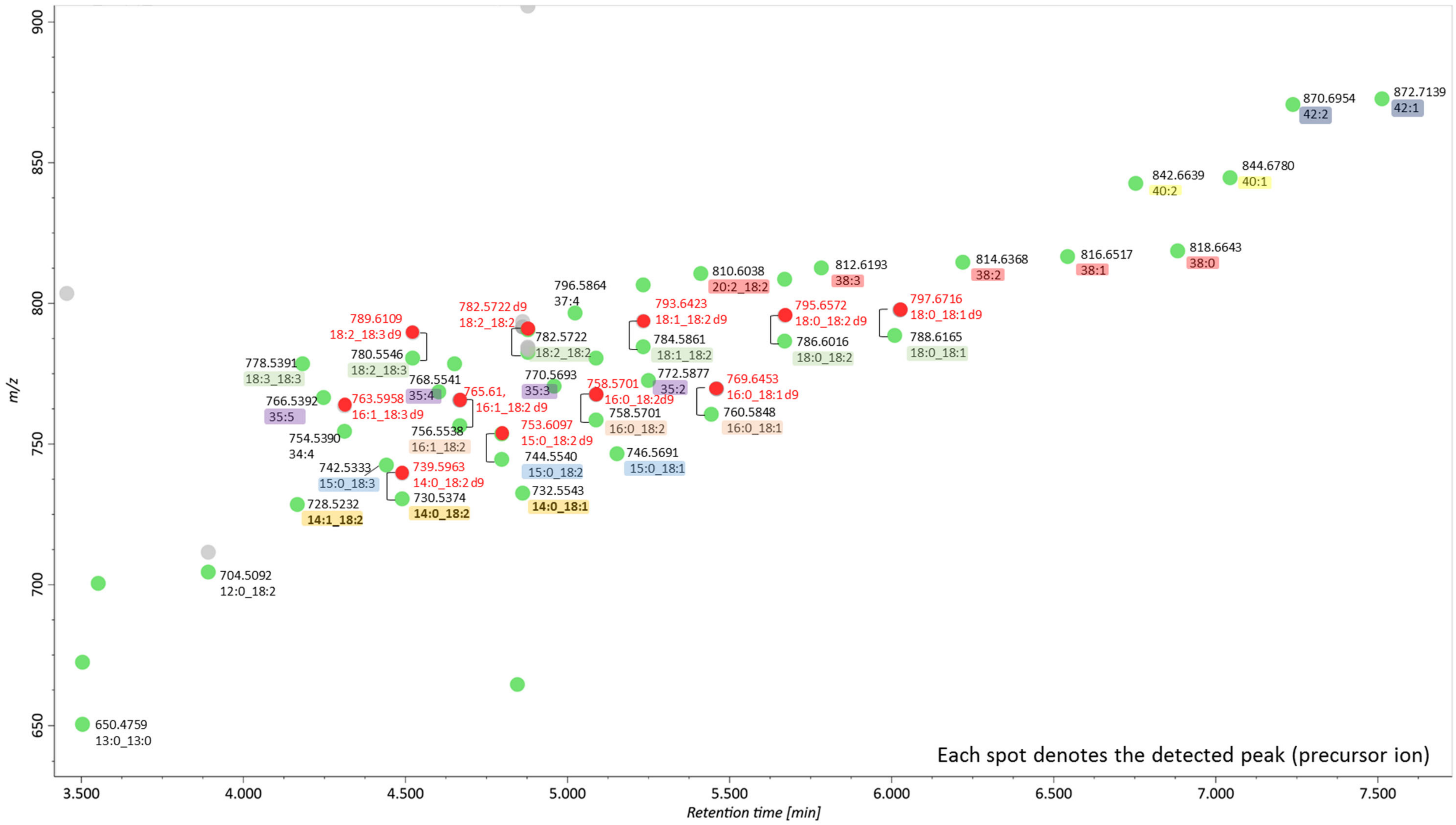

Supplement: Supplementary file 1 [file jof-09-00102-s001.zip › Figure S2. LC-MS.pdf]
