# Supplementary material for: Heterogeneous Distribution of Phospholipid Molecular Species in the Surface Culture of Flammulina velutipes: New Facts about Lipids Containing α-Linolenic Fatty Acid
Source: J Fungi (Basel). 2023 Jan 12;9(1):102. doi: 10.3390/jof9010102 (PMC9865325; doi:10.3390/jof9010102)
Supplement: Supplementary file 1 [file jof-09-00102-s001.zip › Table S1. SWATH settings.pdf]

Table S1. SWATH settings

Event#	+/-	Type	Start (min)	End (min)	Precursor Ion m/z	TOF Start m/z	TOF End m/z	CE	CE Spread (+/-)	Event Time (s)	Q1 Resolution	Interface Voltage (kV)
1	+	MS	2	11		640	1050			0.1		4
2	+	MS/MS	2	11	647.05	150	1050	30	5	0.03	14.1	4
3	+	MS/MS	2	11	661.15	150	1050	30	5	0.03	14.1	4
4	+	MS/MS	2	11	675.25	150	1050	30	5	0.03	14.1	4
5	+	MS/MS	2	11	689.35	150	1050	30	5	0.03	14.1	4
6	+	MS/MS	2	11	703.45	150	1050	30	5	0.03	14.1	4
7	+	MS/MS	2	11	717.55	150	1050	30	5	0.03	14.1	4
8	+	MS/MS	2	11	731.65	150	1050	30	5	0.03	14.1	4
9	+	MS/MS	2	11	745.75	150	1050	30	5	0.03	14.1	4
10	+	MS/MS	2	11	759.85	150	1050	30	5	0.03	14.1	4
11	+	MS/MS	2	11	773.95	150	1050	30	5	0.03	14.1	4
12	+	MS/MS	2	11	788.05	150	1050	30	5	0.03	14.1	4
13	+	MS/MS	2	11	802.15	150	1050	30	5	0.03	14.1	4
14	+	MS/MS	2	11	816.25	150	1050	30	5	0.03	14.1	4
15	+	MS/MS	2	11	830.35	150	1050	30	5	0.03	14.1	4
16	+	MS/MS	2	11	844.45	150	1050	30	5	0.03	14.1	4
17	+	MS/MS	2	11	858.55	150	1050	30	5	0.03	14.1	4
18	+	MS/MS	2	11	872.65	150	1050	30	5	0.03	14.1	4
19	+	MS/MS	2	11	886.75	150	1050	30	5	0.03	14.1	4
20	+	MS/MS	2	11	900.85	150	1050	30	5	0.03	14.1	4
21	+	MS/MS	2	11	914.95	150	1050	30	5	0.03	14.1	4
22	+	MS/MS	2	11	929.05	150	1050	30	5	0.03	14.1	4
23	+	MS/MS	2	11	943.15	150	1050	30	5	0.03	14.1	4
24	+	MS/MS	2	11	957.25	150	1050	30	5	0.03	14.1	4
25	+	MS/MS	2	11	971.35	150	1050	30	5	0.03	14.1	4
26	+	MS/MS	2	11	985.45	150	1050	30	5	0.03	14.1	4

27	+	MS/MS	2	11	999.55	150	1050	30	5	0.03	14.1	4
28	+	MS/MS	2	11	1013.65	150	1050	30	5	0.03	14.1	4
29	+	MS/MS	2	11	1027.75	150	1050	30	5	0.03	14.1	4
30	+	MS/MS	2	11	1041.85	150	1050	30	5	0.03	14.1	4
