# Supplementary material for: Heterogeneous Distribution of Phospholipid Molecular Species in the Surface Culture of Flammulina velutipes: New Facts about Lipids Containing α-Linolenic Fatty Acid
Source: J Fungi (Basel). 2023 Jan 12;9(1):102. doi: 10.3390/jof9010102 (PMC9865325; doi:10.3390/jof9010102)
Supplement: Supplementary file 1 [file jof-09-00102-s001.zip › Table S2. PC and PE species identified by SWATH MS.pdf]

	$[M+H]^+$ experimental	$[M+H]^+$ theoretical	Delta	RT, min	MS/MS fragments (Detected fragments are shown in bold)					
14:1_18:3 PC	726.5095	726.5074	0.0022	3.84	**184.07**	466.29	518.33	448.28	500.31	
14:1_18:2 PC	728.5222	728.5231	-0.0008	4.17	**184.07**	**468.31**	518.33	450.30	500.31	
14:0_18:2 PC	730.5400	730.5387	0.0014	4.49	**184.07**	466.29	520.34	448.28	502.32	
14:0_18:1 PC	732.5555	732.5543	0.0013	4.86	**184.07**	**468.31**	522.35	**450.30**	504.34	
15:0_18:3 PC	742.5394	742.5387	0.0008	4.43	**184.07**	**482.32**	518.33	464.31	500.31	
15:0_18:2 PC	744.5550	744.5543	0.0008	4.80	**184.07**	**482.32**	**520.34**	**464.31**	**502.32**	
15:0_18:1 PC	746.5714	746.5700	0.0015	5.14	**184.07**	**482.32**	**522.35**	**464.31**	**504.35**	
13:0_13:0 PC	650.4769	650.4761	0.0008	3.49	**184.07**	**454.29**	**454.29**	**436.28**	**436.28**	
34:5* PC	752.5252	752.5231	0.0022	3.96	**184.07**					
34:4 PC	754.5374	754.5387	-0.0012	4.31	**184.07**					
16:1_18:2	756.5557	756.5543	0.0014	4.72	**184.07**	**494.32**	520.34	476.31	**502.32**	
16:0_18:2	758.5701	758.5700	0.0002	5.07	**184.07**	**496.34**	**520.34**	**478.32**	**502.32**	
16:0_18:1 PC	760.5884	760.5856	0.0028	5.44	**184.07**	**496.34**	**522.35**	**478.32**	**504.34**	
35:4 PC	768.5555	768.5544	0.0012	4.59	**184.07**					
18:3_18:3 PC	778.5386	778.5387	0.0000	4.17	**184.07**	518.32	518.33	**500.31**	500.31	
18:2_18:3 PC	780.5543	780.5543	0.0000	4.52	**184.07**	**520.34**	**518.33**	**502.32**	500.31	
18:2_18:2 PC	782.5707	782.5700	0.0007	4.88	**184.07**	**520.34**	**520.34**	**502.32**	**502.32**	
18:1_18:2 PC	784.5854	784.5856	-0.0001	5.23	**184.07**	**522.35**	**520.34**	**504.34**	**502.32**	
18:0_18:2 PC	786.6010	786.6013	-0.0002	5.67	**184.07**	**524.37**	**520.34**	**506.36**	**502.32**	
18:0_18:1 PC	788.6149	788.6169	-0.0019	6.03	**184.07**	**524.37**	**522.35**	**506.36**	**504.34**	
37:4 PC	796.5864	796.5857	0.0008	5.02	**184.07**					
20:2_18:2 PC	810.6038	810.6013	0.0026	5.41	**184.07**	**520.34**	**548.37**	**502.32**	**530.36**	
38:3 PC	812.6195	812.6169	0.0027	5.78	**184.07**					
38:2 PC	814.6335	814.6326	0.0010	6.22	**184.07**					
19:0_19:0 PC	818.6642	818.6633	0.0009	6.87	**184.07**	**538.39**	**538.39**	**520.37**	**520.37**	
38:1 PC	816.6517	816.6482	0.0035	6.54	**184.07**					
40:3 PC	840.6457	840.6482	-0.0025	6.45	**184.07**					
40:2 PC	842.6653	842.6639	0.0015	6.75	**184.07**					
40:1 PC	844.6780	844.6796	-0.0015	7.04	**184.07**					
42:2 PC	870.6954	870.6952	0.0003	7.24	**184.07**					
42:1 PC	872.7139	872.7109	0.0031	7.51	**184.07**					
12:0_18:2 PE	660.4597	660.4604	-0.0007	4.02	**519.44**	**142.03**	257.21	337.27	183.17	263.24
15:0_15:0 PE	664.4940	664.4917	0.0023	4.83	523.4726	**142.03**	440.28	440.28	422.27	422.27
14:0_18:2 PE	688.4962	688.4917	0.0045	4.619	547.4726	**142.03**	**285.24**	**337.27**	**211.21**	**263.24**
14:0_18:1 PE	690.5040	690.5074	-0.0034	4.99	**549.49**	**142.03**	285.24	339.29	211.21	265.25
33:3 PE	700.4940	700.4934	0.0006	4.52	**559.47**	**142.03**	299.26	335.26	225.22	261.22
15:0_18:2 PE	702.4996	702.5074	-0.0078	4.91	**561.49**	**142.03**	**299.26**	**337.27**	**225.22**	**263.24**
34:4 PE	712.4954	712.4917	0.0037	4.41	**571.47**	**142.03**				
16:1_18:2 PE	714.5063	714.5074	-0.0011	4.81	**573.49**	**142.03**	**311.26**	**337.27**	**237.22**	**263.24**
16:0_18:2 PE	716.5256	716.5230	0.0026	5.20	**575.50**	**142.03**	**313.27**	**337.27**	**239.24**	**263.24**
16:0_18:1 PE	718.5395	718.5387	0.0008	5.57	**577.52**	**142.03**	313.27	**339.29**	**239.24**	**265.25**
18:3_18:3 PE	736.4929	736.5074	-0.0145	4.30	**595.49**	**142.03**	335.26	335.26		
18:2_18:3 PE	738.5099	738.5074	0.0025	4.65	**597.49**	**142.03**	**337.27**	**335.26**	**263.24**	**261.22**
18:2_18:2 PE	740.5245	740.5230	0.0015	4.99	**599.50**	**142.03**	**337.27**	**337.27**	**263.24**	**263.24**
18:1_18:2 PE	742.5401	742.5387	0.0014	5.35	**601.52**	**142.03**	**337.27**	**339.29**	**263.24**	**265.25**
36:2 PE	744.5543	744.5543	0.0000	5.77	**603.54**	**142.03**				

* For lipid species without detected fragments the number of carbon atoms and double bonds in the fatty acid side chains listed
