# Supplementary material for: Heterogeneous Distribution of Phospholipid Molecular Species in the Surface Culture of Flammulina velutipes: New Facts about Lipids Containing α-Linolenic Fatty Acid
Source: J Fungi (Basel). 2023 Jan 12;9(1):102. doi: 10.3390/jof9010102 (PMC9865325; doi:10.3390/jof9010102)
Supplement: Supplementary file 1 [file jof-09-00102-s001.zip › Table S3. PC, PE content in 3 zones.pdf]

Table S3. The content of phospholipid molecular species (% of mol. species in the lipid class)

$[M+H]^+$	Lipids	Center	Middle	Peripheral
726.5095	14:1_18:3 PC	-	0.05	-
728.5222	14:1_18:2 PC	-	0.13	-
730.5400	14:0_18:2 PC	0.68	0.52	0.37
732.5555	14:0_18:1 PC	-	0.08	-
742.5394	15:0_18:3 PC	-	0.08	-
744.5550	15:0_18:2 PC	0.85	0.57	0.32*
746.5714	15:0_18:1 PC	-	0.07	-
752.5252	34:5 PC	-	0.05	-
754.5374	34:4 PC	0.43	0.47	0.45
756.5557	16:1_18:2 PC	2.82	2.39	1.66*
758.5701	16:0_18:2 PC	5.72*	4.66	3.50
760.5884	16:0_18:1 PC	0.72	0.67	0.63
768.5555	35:4 PC	0.30	0.26	-
778.5386	18:3_18:3 PC	1.17	1.64	5.17*
780.5543	18:2_18:3 PC	13.74*	18.28*	27.45*
782.5707	18:2_18:2 PC	53.35*	44.76	41.84
784.5854	18:1_18:2 PC	7.32	8.48	7.02
786.6010	18:0_18:2 PC	3.05	3.68	2.82
788.6149	18:0_18:1 PC	0.49	0.68	0.43
796.5864	37:4 PC	0.58	0.50	-
810.6038	20:2_18:2 PC	-	0.14	0.20
812.6195	38:3 PC	-	0.14	0.22
814.6335	38:2 PC	0.24	0.23	0.20
816.6517	38:1 PC	-	0.06	-
840.6457	40:3 PC	-	-	0.09
842.6653	40:2 PC	0.34	0.37	0.33
844.6780	40:1 PC	-	0.10	0.06
870.6954	42:2 PC	0.37	0.34	0.32
872.7139	42:1 PC	-	0.14	0.05
660.4597	12:0_18:2 PE	1.82	1.43	-
688.4962	14:0_18:2 PE	-	1.69	0.94
690.5040	14:0_18:1 PE	-	0.23	-
700.4940	33:3 PE	-	0.25	-
702.4996	15:0_18:2 PE	-	2.88	-
712.4954	34:4 PE	-	0.86	-
714.5063	16:1_18:2 PE	15.90*	9.28*	4.28*
716.5256	16:0_18:2 PE	22.78*	18.49	18.74
718.5395	16:0_18:1 PE	-	1.32	0.91
736.4929	18:3_18:3 PE	-	0.29	1.72*
738.5099	18:2_18:3 PE	4.93*	8.16*	17.14*
740.5245	18:2_18:2 PE	39.33	45.18	40.67
742.5401	18:1_18:2 PE	16.89	13.96	15.29
744.5543	36:2 PE	-	1.2	1.70

* Lipids showing significant differences (p-value < 0.05) between zones as determined by Student's t-test
